# Communication between alveolar macrophages and fibroblasts via the TNFSF12-TNFRSF12A pathway promotes pulmonary fibrosis in severe COVID-19 patients

**DOI:** 10.1186/s12967-024-05381-7

**Published:** 2024-07-29

**Authors:** Lei Guo, Qiong Chen, Mengying Xu, Jing Huang, Hua Ye

**Affiliations:** 1 Department of Infection Control, The Second Affiliated Hospital and Yuying Children’s Hospital of Wenzhou Medical University, Wenzhou, 325000 People’s Republic of China; 2 Department of Neurology, The Wenzhou Third Clinical Institute Affiliated To Wenzhou Medical University, The Third Affiliated Hospital of Shanghai University, Wenzhou People’s Hospital, 299 Gu’an Road, Ouhai District, Wenzhou, 325000 Zhejiang People’s Republic of China

**Keywords:** Severe COVID-19, Pulmonary fibrosis, Alveolar macrophages, Fibroblasts, TNFSF12-TNFRSF12A pathway, Cell communication, Therapeutic strategy

## Abstract

**Background:**

Severe COVID-19 infection has been associated with the development of pulmonary fibrosis, a condition that significantly affects patient prognosis. Understanding the underlying cellular communication mechanisms contributing to this fibrotic process is crucial.

**Objective:**

In this study, we aimed to investigate the role of the TNFSF12-TNFRSF12A pathway in mediating communication between alveolar macrophages and fibroblasts, and its implications for the development of pulmonary fibrosis in severe COVID-19 patients.

**Methods:**

We conducted single-cell RNA sequencing (scRNA-seq) analysis using lung tissue samples from severe COVID-19 patients and healthy controls. The data was processed, analyzed, and cell types were annotated. We focused on the communication between alveolar macrophages and fibroblasts and identified key signaling pathways. In vitro experiments were performed to validate our findings, including the impact of TNFRSF12A silencing on fibrosis reversal.

**Results:**

Our analysis revealed that in severe COVID-19 patients, alveolar macrophages communicate with fibroblasts primarily through the TNFSF12-TNFRSF12A pathway. This communication pathway promotes fibroblast proliferation and expression of fibrotic factors. Importantly, silencing TNFRSF12A effectively reversed the pro-proliferative and pro-fibrotic effects of alveolar macrophages.

**Conclusion:**

The TNFSF12-TNFRSF12A pathway plays a central role in alveolar macrophage-fibroblast communication and contributes to pulmonary fibrosis in severe COVID-19 patients. Silencing TNFRSF12A represents a potential therapeutic strategy for mitigating fibrosis in severe COVID-19 lung disease.

**Supplementary Information:**

The online version contains supplementary material available at 10.1186/s12967-024-05381-7.

## Introduction

Patients with severe coronavirus disease 2019 (COVID-19) may develop pulmonary fibrosis following infection [1]. Pulmonary fibrosis is a condition characterized by the excessive scarring of lung tissue, resulting in reduced lung elasticity and impairment of respiratory function [2]. Treating COVID-19-induced pulmonary fibrosis poses various challenges due to the difficulty in reversing the scarring of lung tissue [3]. Injured lung tissue can result in chronic respiratory discomfort and diminished oxygen exchange efficiency [4]. Patients with pulmonary fibrosis may experience a significant decline in their quality of life and are at risk for disease recurrence and the development of additional complications [5]. From a long-term standpoint, these patients generally have a poor prognosis due to limited treatment options and a high rate of recurrence [6]. In general, the presence of COVID-19-induced pulmonary fibrosis has exacerbated the challenges in treating the disease and has adversely impacted the long-term health and quality of life of patients [7].

Alveolar macrophages play a pivotal role in the immune response within the lungs due to their capability to phagocytose and eliminate pathogens and deceased cells [8]. Recent studies have indicated that the communication mechanism between alveolar macrophages and fibroblasts may significantly contribute to the pathogenesis of pulmonary fibrosis in critically ill patients with COVID-19 [9]. This communication mechanism involves the release of inflammatory mediators, which promote the proliferation of fibroblasts and the secretion of collagen [10]. Excessive fibroblast activity results in the overproduction of scar tissue, thereby leading to the development of pulmonary fibrosis [11]. Hence, understanding the interaction between these cell types could yield novel therapeutic targets to prevent or treat COVID-19-induced pulmonary fibrosis [12].

Preliminary bioinformatics analysis in this study predicts that the TNFSF12-TNFRSF12A pathway may have a significant role in fibroblast communication [13]. TNFSF12 and TNFRSF12A are two proteins that potentially interact and may impact the activity and function of fibroblasts [13]. Previous studies have demonstrated an association between the TNFSF12-TNFRSF12A pathway and pulmonary fibrosis, indicating its significant role in the development and progression of the disease [14]. Based on these two pieces of information, it can be inferred that the TNFSF12-TNFRSF12A pathway may play a crucial role in influencing fibroblast behavior and promoting the progression of pulmonary fibrosis [13]. Thorough investigation of this pathway not only elucidates the mechanisms of pulmonary fibrosis but also facilitates the identification of novel strategies and targets for targeted therapy [15].

This study investigates the interaction between alveolar macrophages and fibroblasts via the TNFSF12-TNFRSF12A pathway. It aims to deepen the understanding of how this mechanism contributes to the development of lung fibrosis in critically ill COVID-19 patients. The aim of this study is to elucidate the molecular mechanisms underlying pulmonary fibrosis in severe cases of COVID-19, with the goal of facilitating the identification of novel treatment approaches and prevention strategies. By gaining a deeper understanding of this interplay mechanism, researchers can not only better comprehend the complications and progression of COVID-19, but also potentially provide more precise treatment options for patients, thus enhancing treatment efficacy and improving the quality of life.

## Materials and methods

### Public database data download

The single-cell RNA sequencing (scRNA-seq) datasets GSE122960 and GSE149878, along with microarray data GSE40839, were acquired from the gene expression omnibus (GEO) repository, accessible at https://www.ncbi.nlm.nih.gov/gds. Comprehensive sample information for all GEO datasets can be found in Table S1. Notably, ethical committee approval was unnecessary, as these datasets originate from a publicly accessible database.

### Quality control of scRNA-seq data

Upon obtaining the expression matrix through the official software Cellranger provided by 10 × Genomics, a series of criteria were applied for cell filtering. Specifically, cells that met the following conditions were retained: a minimum of 200 expressed genes per single cell (nFeature_RNA > 200), a minimum of 1000 detected RNAs per single cell (nCount_RNA > 1000), and a mitochondrial gene percentage of less than 15% (percent.mt < 15%). Subsequently, unique molecular identifiers (UMIs) were utilized for gene correlation analysis to assess the quality of the filtered data. All analyses were performed using R software (version 4.2.3) [16].

### UMAP clustering analysis and cell annotation

The initial step involves conducting a principal component analysis (PCA) on the dataset's top 2000 genes characterized by the highest variance. Following this, the JackStrawPlot and ElbowPlot functions are utilized to determine the most suitable principal components for subsequent analysis. Subsequently, the FindClusters function provided by Seurat is applied to identify prominent cell subgroups, with a resolution parameter set to 0.5. The next phase involves nonlinear dimensionality reduction of the scRNA-seq sequencing data using the UMAP algorithm, resulting in two-dimensional visualization. Finally, we employed the find marker genes function from the scCATCH package to identify marker genes specific to each cell subpopulation. The process of manual annotation for cell types was carried out using established marker genes [17].

### Temporal analysis

In this study, a strategy akin to Monocle was employed to temporally arrange individual cells along a trajectory that corresponds to essential biological processes, such as cellular differentiation. Pseudotime analysis was conducted utilizing the monocle2 package. The overall procedure can be outlined as follows: Initially, high-dimensional data was mapped onto a lower-dimensional space through the utilization of various dimensionality reduction techniques, including principal component analysis (PCA). Subsequently, a tree structure was created from the resulting tree by employing the DDRTree algorithm, which automatically selected data centroids. The algorithm involved the relocation of cells to their nearest tree vertices, adjustments to vertex positions to accommodate the cells, and the establishment of a new spanning tree. This iterative process continued until alignment between the tree structure and cells was achieved. Following this, pseudotime was computed for each cell by measuring the geodesic distance along the tree to the root. Consequently, branches were automatically assigned to each cell based on the principal plot, thereby enabling a comprehensive analysis of cellular temporal progression [18].

The general process of pseudotime analysis involves the following steps: creating a CellDataSet, specifying the expression family parameter in newCellDataSet, estimating size factors and dispersions, selecting genes that define the trajectories, performing dimensionality reduction, constructing and visualizing pseudotime trajectories, plotting the skeleton diagram of the trajectories, coloring them based on cell types and pseudotime, displaying different branches or states, and observing the gene expression changes along the pseudotime [18].

### Cell communication analysis

CellPhoneDB is a comprehensive database encompassing a wide array of ligands, receptors, and their intricate interactions. It serves as a valuable resource for conducting in-depth analyses of intercellular communication molecules. This database facilitates the exploration of cross-talk and communication networks that underlie the interactions between diverse cell types, thereby shedding light on intricate cellular communication mechanisms [19].

In this particular study, the researchers employed the R software package known as CellChat [20]. Leveraging data retrieved from the CellPhoneDB database, the investigators conducted a rigorous analysis of cellular communication, focusing on three key aspects: Secreted Signaling, ECM-Receptor, and Cell–Cell Contact.

The overall workflow for this analysis entails a sequence of critical steps. Initially, a CellChat object is instantiated, drawing upon prior scRNA-Seq data. Subsequently, the trimean method is employed to estimate the number of ligand-receptor pairs, a crucial aspect of the analysis. Following this, the communication probabilities associated with all ligand-receptor interactions within each signaling pathway are amalgamated, contributing to the calculation of communication probabilities at the signaling pathway level. These pathways are subsequently visualized through a variety of representations, including hierarchy networks, circle plots, and chord diagrams. Finally, the significance of individual signaling pathways is evaluated and ranked based on disparities in the overall information flow within the network between the two distinct groups of interest, providing valuable insights into the essentiality of these pathways [20].

### Scoring the severity of disease caused by SARS-CoV-2

To obtain information pertaining to “SARS-CoV-2,” a search was conducted within the GeneCards database (https://www.genecards.org/) by specifying a relevance score threshold greater than 20. Following this initial query, the SARS-CoV-2 score was computed. This score was determined by employing the AUCell package, and the ensuing score disparities were visualized across various cellular contexts [21].

### Gene differential expression analysis

The GSE40839 dataset [22] was subjected to analysis using the R software package limma. This study's objective was to investigate the differentially expressed genes between two groups: normal control lung fibroblasts and lung fibrotic tissue fibroblasts. The selection criteria for these genes involved considering fibroblast samples with an absolute log-fold change exceeding 1 and a P-value below 0.05. Additionally, a separate differential analysis was performed on alveolar macrophages from both the HC (Healthy Control) and SC (Smoking Control) groups. To identify genes with distinctive expression patterns, a threshold of |avg_log2FC|> 0.5 and P < 0.05 was applied during the analysis.

### Gene functional enrichment analysis

To gain a more comprehensive understanding of the functional distinctions between the alveolar macrophages of the HC (Healthy Control) group and SC (Smoking Control) group, we employed the clusterProfiler package in the R software [23]. A significance threshold of P < 0.05 was set, and gene ontology (GO) and Kyoto Encyclopedia of Genes and Genomes (KEGG) enrichment analyses were conducted on the specific gene expression profiles of alveolar macrophages from both the HC and SC groups. The GO analysis covered biological processes (BP), cellular components (CC), and molecular functions (MF), allowing us to explore various aspects of gene function. Additionally, KEGG enrichment analysis was performed on the differentially expressed genes within the GSE40839 dataset to identify potential pathways that may be associated with the observed differences between the two groups.

### Cell culture

The MH-S cell line, which originates from murine alveolar macrophages, was maintained in F12K medium supplemented with 10% fetal bovine serum and 1% penicillin–streptomycin at a temperature of 37℃ within a 5% CO2 incubator [24]. These MH-S cells were subjected to treatment with 1 μg/mL of SARS-CoV-2-E (NBP2-90986, Novus, USA) for a duration of 24 h [25].

For the mouse lung fibroblasts (CP-M006, Wuhan Punois Life Science Co., Ltd.), they were cultured in Dulbecco's Modified Eagle Medium (DMEM) supplemented with 10% fetal bovine serum and 1% penicillin–streptomycin at 37 °C within a 5% CO_2_ incubator [26].

In subsequent experiments, MH-S alveolar macrophages treated with SARS-CoV-2-E for 24 h were placed in the upper chamber (well3422, Corning) of Transwell plates at a density of 105 cells. Simultaneously, fibroblast cells isolated from mouse lungs were seeded into the lower chamber of the Transwell plates. These two distinct cell types were co-cultured for a period of 48 h, following which both the supernatant from the cell culture and the fibroblast cells themselves were collected for further experimentation.

### Lentivirus infection and grouping

Mouse lung fibroblast cells, which were in the logarithmic growth phase, were cultured and detached using trypsin, resulting in a cell suspension with a concentration of 5 × 104 cells/mL. Subsequently, 2 mL of this cell suspension was seeded into each well of a 6-well plate, and the corresponding lentivirus was added. The lentivirus had a titer of 1 × 109 TU/mL, and the multiplicity of infection (MOI) for infecting cells was set at 10. After a 48-h intervention period, cells were collected for subsequent testing.

The cell groups used in this study consisted of the following:

oe-NC: Cells infected with an overexpressed control lentivirus.

oe-TNFSF12: Cells infected with an overexpressed TNFSF12 lentivirus.

oe-TNFRSF12A: Cells infected with an overexpressed TNFRSF12A-1 lentivirus.

sh-NC: Cells infected with a silenced control lentivirus carrying the sequence 5ʹ-TTCTCCGAACGTGTCACGTTTC-3ʹ [27].

sh-TNFRSF12A-1: Cells infected with a TNFRSF12A silencing lentivirus using the sequence 5ʹ-CCGGTCGTCGTCCATTCATTCATTCCTCGAGGAATGAATGAATGGACGACGATTTTTG-3ʹ.

sh-TNFRSF12A-2: Cells infected with a TNFRSF12A-2 silencing lentivirus using the sequence 5ʹ-CCGGACTAAGGAACTGCAGCATTTGCTCGAGCAAATGCTGCAGTTCCTTAGTTTTTTG-3ʹ.

Here, "NC" denotes the negative control, and “oe” signifies overexpression. All lentiviruses were procured from Shanghai Jima Pharmaceutical Technology Co., Ltd. Following 48 h of infection, the culture medium was replaced to prepare for subsequent experiments. This experiment was repeated three times on separate occasions [28].

### CCK-8

During the logarithmic growth phase, mouse lung fibroblast cells were plated in a 96-well plate at a density of 5000 cells per well. Subsequently, 10 μL of CCK-8 reagent solution (C0038, Shanghai Biyun Tian Biotechnology Co., Ltd) was added to each well. The plate was then placed in a humidified incubator at 37 ℃. After incubating for one hour, the absorbance at 450 nm was measured using the Epoch Microplate Spectrophotometer (Bio-Tek, Winooski, VT, USA). This procedure was performed with three replicates for each sample [29].

### ELISA

The culture medium from murine alveolar macrophages (MH-S) or the co-culture solution of MH-S and murine lung fibroblasts (Fibroblast) was subjected to centrifugation at 1500×*g* for 15 min. The quantification of TNFSF12 levels was conducted according to the procedural guidelines outlined in the mouse TNFSF12 ELISA kit manual (RAB0495, Sigma-Aldrich). Subsequently, the absorbance at 450 nm was measured utilizing the Epoch model on a microplate spectrophotometer (Bio-Tek, Winooski, VT, USA). For each sample, three replicate measurements were taken [30].

### RT-qPCR

Total RNA was extracted from cells using TRIzol (15596026, Invitrogen, Carlsbad, CA, USA). The concentration and purity of the extracted total RNA were assessed using a NanoDrop 2000 spectrophotometer (Model 1011U, NanoDrop, USA). Subsequently, the mRNA was reverse transcribed into cDNA following the protocols outlined in the PrimeScript RT reagent Kit (RR047A, Takara, Japan). Real-time quantitative PCR was performed utilizing the ABI7500 quantitative PCR instrument (7500, ABI, USA). The PCR reaction conditions involved an initial denaturation at 95 °C for 10 min, followed by denaturation at 95 °C for 10 s, annealing at 60 °C for 20 s, and extension at 72 °C for 34 s, repeated for a total of 40 cycles. GAPDH served as an internal reference. The relative transcription level of the target gene was determined using the comparative quantification method (2-ΔΔCT method). This involved the calculation of the ΔΔCt value, obtained by subtracting the ΔCt of the control group from the ΔCt of the experimental group. The ΔCt represents the Ct value of the target gene minus the Ct value of the reference gene. Finally, the relative transcription level of the target gene was calculated as 2−ΔΔCt [31]. Each experiment was performed in triplicate, and the primers were synthesized by TaKaRa Company (Table 1).

**Table 1:** RT-qPCR Primer sequence

Gene	Primer sequence(5ʹ-3ʹ)
TNFSF12	F: 5ʹ-AAGTTCACTGAGGGGCCTTG-3ʹ
	R: 5ʹ-TAAACACGTGGTCGGGTGAG-3ʹ
TNFRSF12A	F: 5ʹ-CAATCATGGCTTCGGCTTGG-3ʹ
	R: 5ʹ-GCGCCTGGTGCTTGCT-3ʹ
αSMA	F: 5ʹ-GTACCCAGGCATTGCTGACA-3ʹ
	R: 5ʹ-GCTGGAAGGTAGACAGCGAA-3ʹ
Collagen I	F: 5ʹ-GCCCGAACCCCAAGGAAAAGAAGC-3ʹ
	R: 5ʹ-CTGGGAGGCCTCGGTGGACATTAG-3ʹ
Fibronectin	F: 5ʹ-ATGAGAAGCCTGGATCCCCT-3ʹ
	R: 5ʹ-GGAAGGGTAACCAGTTGGGG-3ʹ
GAPDH	F: 5ʹ-GGAGAGTGTTTCCTCGTCCC-3ʹ
	R: 5ʹ-ACTGTGCCGTTGAATTTGCC-3ʹ

F: Forward; R: Reverse

### Western blot

To lyse and extract total protein from cells, RIPA lysis buffer (P0013B, Beyotime, Shanghai) containing PMSF was employed, and protein quantification was performed using the BCA protein analysis kit (23225, Thermo Fisher Scientific, Rockford, IL, USA). Specifically, 50 μg of protein was dissolved in 2 × SDS loading buffer and subsequently boiled at 100 °C for 5 min. The samples were then subjected to SDS-PAGE gel electrophoresis. Protein transfer from the gel to a PVDF membrane was achieved using the wet transfer method. Subsequently, the PVDF membrane was blocked with 5% skim milk at room temperature for 1 h. Primary antibodies, including Rabbit anti-α-SMA (1:1000, ab5694, Abcam), Collagen I (1:1000, ab270993, Abcam), Fibronectin (1:1000, ab2413, Abcam), and GAPDH (1:1000, ab9485, Abcam), were diluted and incubated with the PVDF membrane overnight at 4 °C. The membrane was subjected to TBST washes, each lasting for 10 min. Following this, the membrane was incubated with the secondary antibody, Goat Anti-Rabbit IgG H&L (HRP) (ab97051, 1:2000, Abcam, Cambridge, UK), for 1 h. Subsequent TBST washes were performed, and the membrane was placed on a clean glass plate. A mixture of Solution A and Solution B from the Pierce™ ECL Detection Kit (Catalog number 32209, Thermo) was evenly applied to the membrane in a dark room. The protein bands were visualized using the Bio-Rad Imaging System (ChemiDoc™ XRS + , BIO-RAD Company, USA) [31]. Grayscale values of each band were analyzed using the gel image analysis software ImageJ, and the ratio between the grayscale values of the target protein and the internal reference protein bands was calculated. This experiment was repeated three times.

### Statistical analysis

All data were analyzed utilizing SPSS 21.0 Statistical Software (SPSS, Inc., Chicago, IL, USA). The measurement data are presented as mean ± standard deviation. Comparisons between two groups were conducted using an unpaired t-test, whereas comparisons among multiple groups were carried out using one-way ANOVA. Statistical significance was determined when P < 0.05.

## Results

### Single-cell transcriptomic analysis reveals predominance and dysregulation of myeloid cells in severe COVID-19 lung tissue

The worldwide pandemic caused by SARS-CoV-2, which has led to the emergence of the COVID-19, has resulted in millions of confirmed cases and fatalities globally. In some patients, infection with SARS-CoV-2 could lead to severe respiratory failure, necessitating mechanical ventilation. This condition is called Acute Respiratory Distress Syndrome (ARDS) and is commonly known as severe COVID-19 [32]. The lungs serve as the primary target organs for SARS-CoV-2. Nonetheless, our comprehension of how SARS-CoV-2 infection contributes to lung pathology at the cellular and molecular levels remains limited. Hence, analyzing the single-cell traits of lung tissue samples from critically ill COVID-19 patients utilizing single-cell RNA sequencing (scRNA-seq) technology represents a sophisticated and practicable approach [33, 34].

To create a comprehensive single-cell transcriptomic atlas of lung tissue in critically ill COVID-19 patients, we acquired severe COVID-19-related single-cell RNA sequencing (scRNA-seq) data from the GSE149878 dataset in the GEO database. This dataset comprised lung tissue samples (SC group) from four critically ill COVID-19 patients. Furthermore, we obtained four healthy control lung tissue samples (referred to as the HC group) from the GSE122960 dataset.

Firstly, the data was subjected to quality control and normalization using the R software package Seurat. Ultimately, a total of 31,841 single cells in the SC group and 22,085 single cells in the HC group passed the quality control process (Figure S1A, D). The correlation calculation results for sequencing depth indicate a negative correlation between the filtered data nCount_RNA and percent.mt (r = − 0.03 and r = − 0.09, respectively), while there is a positive correlation between nCount_RNA and nFeature_RNA (r = 0.90 and r = 0.95, respectively) (Figure S1B, E). These results suggest that the filtered cell data exhibits good quality. Lastly, the filtered cells were examined to identify highly variable genes, and a subset of the top 2000 highly variable genes was chosen for further analysis (Figure S1C, F).

Furthermore, we calculated the cell cycle of each sample using the CellCycleScoring function and observed that the cell cycle was consistently similar across various samples (Figure S2A). After removing the batch effect, all samples were combined, and principal component analysis (PCA) was performed on the top 2000 highly variable genes using the RunPCA function. No visible batch effect was observed among the 8 samples, indicating their suitability for further analysis (Figure S2B). To visualize the first 2 principal components, we used the DimHeatmap function to create a heatmap (Figure S2C). Additionally, we identified and displayed the major component genes within these principal components (Figure S2D).

The first 50 principal components were visualized using the JackStrawPlot function. By comparing the position of the P-value distribution of each principal component with respect to the mean distribution, it was revealed that the P-values of the first 12 principal components were all found to be less than 0.05 (Figure S2E). Furthermore, when combined with the ElbowPlot function, it was discovered that the standard deviation at the 12th principal component exhibited a turning point (Figure S2F).

The results above indicate that the initial 12 principal components effectively reflect the information in the selected highly variable genes and carry considerable analytical significance. Thus, we will employ these 12 principal components for UMAP clustering analysis in subsequent steps.

**Fig. 1:**
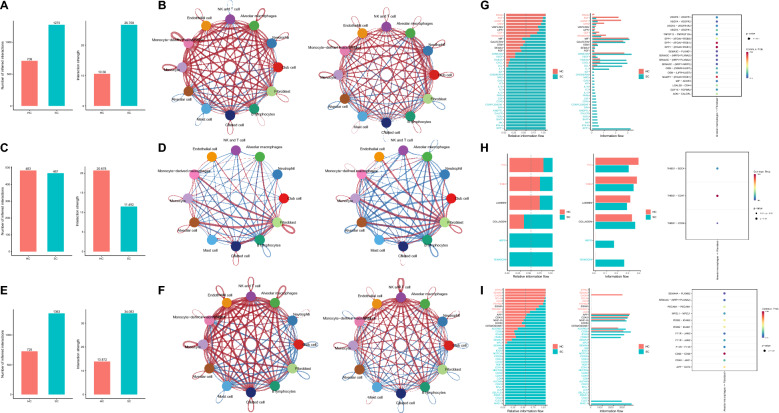
Single-cell atlas of lung tissue samples from severe COVID-19 patients and healthy controls. **A** Based on the expression of known marker genes, 20 cell clusters were annotated into 5 major cell types. **B** Cell clustering and annotation results were visualized to demonstrate the aggregation and distribution of cells in healthy control lung tissue samples (HC group, n = 4) and severe COVID-19 patient lung tissue samples (SC group, n = 4). **C** Bubble plots of cell marker gene expression were used, with darker colors indicating higher average gene expression levels and larger circles indicating a higher number of cells expressing that gene. **D** Cell clustering and annotation results were visualized to illustrate the aggregation and distribution of cells in healthy control lung tissue samples (HC group, n = 4) and severe COVID-19 patient lung tissue samples (SC group, n = 4). **E** Violin plots were used to present the scores of 12 cell SARS-CoV-2 evaluations

Subsequently, the UMAP algorithm was applied to non-linearly reduce the dimensionality of the initial 12 principal components, resulting in the clustering of all cells into 20 cell clusters (Figure S2G). The final annotation was categorized into five major cell types based on cell marker genes: Myeloid cell, Lymphocytes, Epithelial cell, Endothelial cell, and Fibroblast. Among these cell types, Myeloid cells exhibited the highest abundance (Fig. 1A). This study examines the cellular aggregation and distribution of lung tissue samples obtained from healthy control individuals and severe COVID-19 patients. Figure 1B demonstrates a notable variation in Myeloid cell levels between the HC and SC groups. Existing evidence indicates that the dysregulation of Myeloid cells, both locally and systemically, plays a crucial role in exacerbating the severity of COVID-19. Therefore, controlling the quantity and modulating the activity of Myeloid cells could potentially serve as an effective therapeutic approach for treating severe inflammatory lung disease [35].

We generated a single-cell transcriptomic atlas of severe COVID-19 patients and normal lung tissue samples from control subjects. The atlas consists of 53,926 cells obtained using scRNA-seq. Among the recorded cells, the Myeloid cell population exhibited the highest abundance and changes, implying a potentially crucial role in the disease progression of severe COVID-19 patients.

### Characterization and differential analysis of alveolar macrophages highlight their pivotal role in SARS-CoV-2 infection and COVID-19 progression

Based on cell marker genes, we further classified the 5 major cell populations into 12 distinct cell types: Neutrophil, NK cell, T cell, Alveolar macrophages, Alveolar cell, Monocyte, Club cell, Monocyte-derived macrophages, Endothelial cell, Mast cell, B lymphocytes, Ciliated cell, and Fibroblast (Figure S2H).

The expression of marker genes for each cell type is depicted in Fig. 1C. AGER and SFTPC are markers for alveolar cells, while SCGB3A2 and SCGB1A1 function as markers for club cells. TPPP3 and FOXJ1 are indicative of ciliated cells. CD3D, KLRC1, and KLRF1 are markers for NK and T cells, and CD79A, CD79B, and IGHD are markers for B lymphocytes. COL1A1, COL1A2, and DCN are markers for fibroblasts, PECAM1 and VWF are markers for endothelial cells, and CD68 and CD163 serve as markers for macrophages, which could be further categorized into monocyte-derived macrophages (SPP1 and MRC1) and alveolar macrophages (APOC1, APOE, FABP4, MARCO). CD14 is a monocyte marker, and CSF3R is a neutrophil marker. Lastly, TPSB2 is a marker for mast cells. Moreover, HBB serves as the marker gene specific to red blood cells. Consequently, we have excluded cluster 15, representing this particular cell type.

Figure S2H highlights the clear division of alveolar macrophages into two regions. By comparing the cell aggregation and distribution patterns in lung tissue samples from healthy control individuals and severe COVID-19 patients, we observed that Alveolar macrophages in the HC and SC groups originated from two distinct regions (Fig. 1D). This finding indicates a difference in Alveolar macrophages between the two groups.

**Fig. 2:**
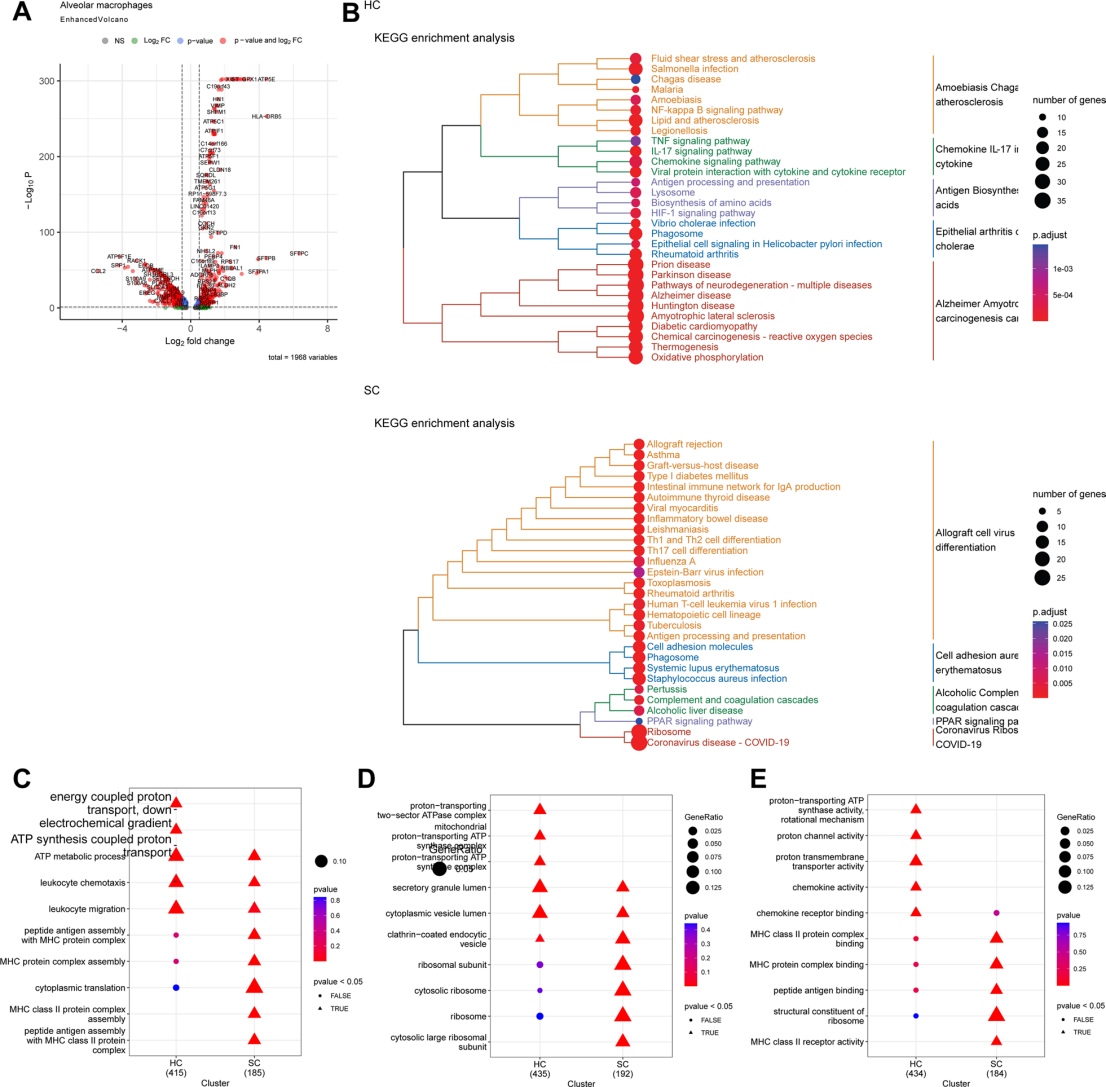
Functional enrichment analysis of differentially expressed genes in alveolar macrophages from lung tissue samples of severe COVID-19 patients and healthy controls. **A** Volcano plot depicting differentially expressed genes in Alveolar macrophages between healthy control lung tissue samples (HC group, n = 4) and severe COVID-19 patient lung tissue samples (SC group, n = 4), with red dots to the left of the dashed line representing genes specifically expressed in the HC group and red dots to the right representing genes specifically expressed in the SC group; **B** KEGG pathway analysis dendrogram of genes specifically expressed in HC and SC groups, categorized into 5 major classes, with each color representing a class; **C** enrichment analysis of Biological Processes (BP) terms for genes specifically expressed in HC and SC groups; **D** enrichment analysis of Cellular Component (CC) terms for genes specifically expressed in HC and SC groups; **E** enrichment analysis of Molecular Function (MF) terms for genes specifically expressed in HC and SC groups

We conducted a differential analysis on alveolar macrophages in the HC and SC groups using a threshold of |avg_log2FC|> 0.5 and adjP < 0.05. We identified a total of 469 genes that were specifically expressed in the HC group of alveolar macrophages and 263 genes that were specifically expressed in the SC group of alveolar macrophages. These results are shown in Fig. 2A and Table S2. Functional enrichment analysis was then conducted on the two groups of genes with distinct expressions. The subsequent KEGG pathway analysis indicated that the SC group exhibited enrichment in virus infection, inflammation, and signal pathways associated with COVID-19 compared to the HC group (Fig. 2B). The results of the GO entry analysis indicated alterations in the functions and components of the SC group MHC class II protein complex binding (Fig. 2C–E). The results above indicate that the quantity and antigen-presenting function of alveolar macrophages in lung tissue samples from critically ill COVID-19 patients has changed, aligning with previously published literature [36].

Furthermore, we obtained 23 genes from the GeneCards database by searching for “SARS-CoV-2” with a Relevance score threshold > 20 (Table S3). Using these genes, we computed AUCell scores for 12 different cell types. The findings revealed that Alveolar macrophages exhibited relatively higher scores than Neutrophils, monocytes, Endothelial cells, and Fibroblasts (Fig. 1E). This finding further underscores the role of alveolar macrophages in both SARS-CoV-2 infection and the progression of COVID-19.

### Pseudo-temporal analysis suggests alveolar macrophages in severe COVID-19 patients potentially originate from monocytes

**Fig. 3:**
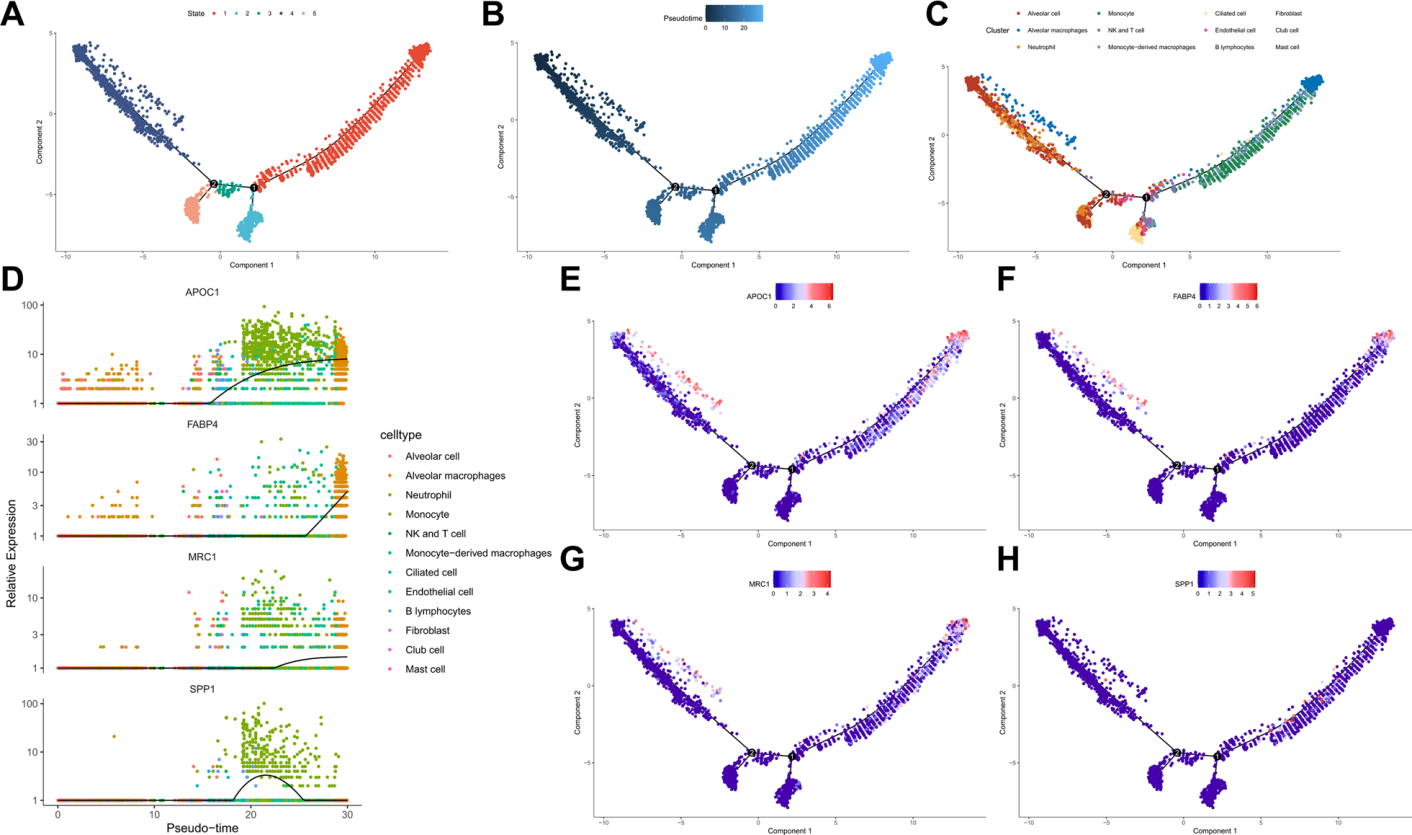
Pseudotemporal analysis of scRNA-seq data from lung tissue samples of severe COVID-19 patients. **A** Trajectory skeleton plots, with each branch (State) displayed separately; **B** trajectory skeleton plots with pseudotime coloring; **C** trajectory skeleton plots with cell type coloring; **D** expression changes of APOC1, FABP4, MRC1, and SPP1 in pseudotime; **E**–**H** trajectory skeleton plots showing the expression changes of APOC1, FABP4, MRC1, and SPP1 in pseudotime

To explore the differentiation of alveolar macrophages in lung tissue samples from severe COVID-19 patients, we performed pseudo-temporal analysis on single-cell RNA sequencing (scRNA-seq) data using the R package monocle2. Our approach involved reducing data dimension using DDRTree, sorting cells based on key gene expression patterns and constructing cell trajectories. Cell evolution is classified into five branches based on the state, with two branch nodes (Fig. 3A).

Pseudotime is a probability calculated by monocle2 using cell gene expression information, representing the temporal order. The left side represents the root of the tree, while the right side represents the branches (Fig. 3B). Our findings show that alveolar macrophages are predominantly localized at the distal ends (Fig. 3C). Considering the trend changes in marker gene expression, it is evident that Monocytes exhibit activity in the latter stages of the pseudo time series, subsequently followed by Monocyte-derived macrophages and ultimately by Alveolar macrophages (Fig. 3D–H).

It is speculated that alveolar macrophages in the lung tissue of patients with severe COVID-19 may originate from monocytes.

### Enhanced inter-cellular communication in severe COVID-19 lung tissue: emphasis on alveolar macrophages and fibroblast interactions

There is mounting evidence to suggest that alveolar macrophages play a role in pulmonary inflammation and fibrosis. These macrophages, derived from monocytes, drive the development of lung fibrosis and persist in the pulmonary tissue [37]. However, the specific mechanism is not yet fully understood. We conducted a detailed investigation into the specific process of communication between alveolar macrophages and other cells in lung tissue samples from patients with severe COVID-19.

The CellChatDB in humans involves 1939 validated molecular interactions, primarily composed of three types of related signaling pathways (Figure S3A). In this study, we conducted cellular communication analysis using single-cell transcriptomic data obtained from severe COVID-19 patients and healthy control lung tissue samples, which were previously constructed. Specifically, we examined cell communication across three aspects: Secreted Signaling, ECM-Receptor, and Cell–Cell Contact.

**Fig. 4:**
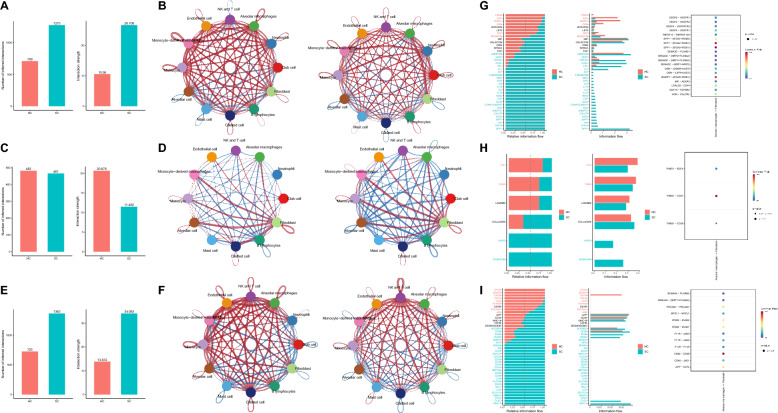
Cellular communication analysis in lung tissue samples from severe cOVID-19 patients and healthy controls. **A**, **C**, and **E** depict bar graphs comparing the number of cell interactions (left) and interaction strength (right) between lung tissue samples from healthy controls (HC group, n = 4) and severe COVID-19 patients (SC group, n = 4), with red indicating more interactions and stronger interactions in the HC group, while blue indicates more interactions and stronger interactions in the SC group. **A** Secreted Signaling; **C** ECM-Receptor; **E** Cell–Cell Contact. **B**, **D**, and **F** display circle plots comparing the number of cell interactions (left) and interaction strength (right) between lung tissue samples from HC and SC groups, with red lines indicating more interactions and stronger interactions in the SC group, and blue lines indicating fewer interactions and weaker interactions in the SC group, with thicker lines signifying more cell interactions. **B** Secreted Signaling; **D** ECM-Receptor; **F** Cell–Cell Contact. **G**–**I** Left panels present bar graphs depicting differential cell communication pathways analysis between lung tissue samples from healthy controls (HC group, n = 4) and severe COVID-19 patients (SC group, n = 4). Red indicates pathways upregulated in the HC group, while blue indicates pathways upregulated in the SC group. The right panels illustrate bubble plots for differential cell communication pathways between Alveolar macrophages and Fibroblasts, with differences highlighted within black dashed boxes. **G** Secreted Signaling; **H** ECM-Receptor; **I** Cell–Cell Contact

Firstly, we compared the total number and strength (weight) of cell interactions in two groups about Secreted Signaling. The results revealed a difference in cell connection tightness between the SC and HC groups. Specifically, the SC group demonstrated cell interaction strength approximately 20 times greater than the HC group (Fig. 4A). Moreover, the alterations in cellular communication between the SC and HC groups were further elucidated through circle plots and heatmaps. Red coloration in these plots signifies upregulation, whereas blue coloration signifies downregulation. The findings indicated that the SC group demonstrated a higher overall number of cell interactions and greater intensity of interactions. Among these interactions, Alveolar macrophages showed a particularly strong communication with other cells, particularly with Fibroblast (Fig. 4B; Figure S3B).

Furthermore, about the ECM-Receptor, there was no difference in the total number of cell interactions observed between the SC group and the HC group. Nevertheless, the intensity of these interactions was lower in the SC group when compared to the HC group (Fig. 4C). In contrast, Fibroblast cells showed more frequent and tighter connections with other cells (Fig. 4D; Figure S3C). Regarding Cell–Cell Contact, the SC group exhibits a greater overall number of cell interactions and stronger interaction strength when compared to the HC group (Fig. 4D). While NK and T cells are likely playing a dominant role in this phenomenon, it is also notable that Alveolar macrophages have a more pronounced contact with Fibroblasts (highlighted by a black dashed box) (Fig. 4E, F; Figure S3D). The above findings suggest severe COVID-19 patients exhibit alterations in the total number and intensity of cell interactions within lung tissue samples compared to healthy control samples. Furthermore, communication among cells within the lung tissue of severe COVID-19 patients primarily occurs through Secreted Signaling and Cell–Cell Contact.

According to the literature, alveolar macrophages specifically accumulate in fibrotic regions near fibroblasts, where they could promote fibroblast proliferation by expressing specific molecules. This mechanism might play a crucial role in their involvement in pulmonary fibrosis. Hence, our study will investigate the interaction between alveolar macrophages and fibroblasts.

### Interplay between alveolar macrophages and fibroblasts: identification of TNFRSF12A as a potential key factor in pulmonary fibrosis

We compared the noteworthy differences in Secreted Signaling, ECM-Receptor, and Cell–Cell Contact between the HC and SC groups. The left figure displays the pathways upregulated in the HC or SC group, represented by red and blue colors. For instance, the Secreted Signaling pathways upregulated in the HC group include PROS, EGF, UGRP1, GRN, GDF, and RESISTIN. On the other hand, the paracrine/autocrine signaling pathways that showed upregulation in the SC group include ANNEXIN, VEGF, TWEAK, CXCL, CCL, IL1, IGF, CHEMERIN, NRG, GAS, KIT, IL16, IL2, IL10, PARs, CSF, CD40, COMPLEMENT, SAA, ANGPTL, CALCR, TGFb, BAFF, MK, IFN-II, and SPP1 (Fig. 4G).

We set ‘sources.use’ to “Alveolar macrophages” and ‘targets.use’ to “Fibroblast”. We present the specific pathways through which Alveolar macrophages interact with Fibroblasts and then perform a screening to identify ligand and receptor factors that exhibit differential expression between these two groups. Using Secreted Signaling as an example, alveolar macrophages could influence fibroblasts by secreting VEGFA, TNFSF12, SPP1, GDF15, and ADM. These molecules bind to VEGFR1, VEGFR2, VEGFR1R2, TNFRSF12A, ITGAV, ITGA5, ITGB5, ITGB1, CD44, TGFBR2, and CALCRL (depicted by the black dashed box), thereby impacting their biological functions (Fig. 4G).

Similarly, alveolar macrophages could interact with fibroblasts in the context of ECM-Receptor through the expression of THBS1 and binding to SDC4, CD47, and CD36 on the surface of fibroblasts (Fig. 4H). Regarding Cell–Cell Contact, Alveolar macrophages could establish interactions with Fibroblasts mediated by the expression of several proteins. The Alveolar macrophages express SEMA4A, PECAM1, MPZL1, ITGB2, F11R, CD99, and CD46, while Fibroblasts express PLXNB2, PLXNA2, PECAM1, MPZL1, ICAM2, ICAM1, JAM3, JAM2, F11R, CD99, and JAG1 (Fig. 4I). To date, we have screened 25 fibroblast receptor factors for further analysis.

**Fig. 5:**
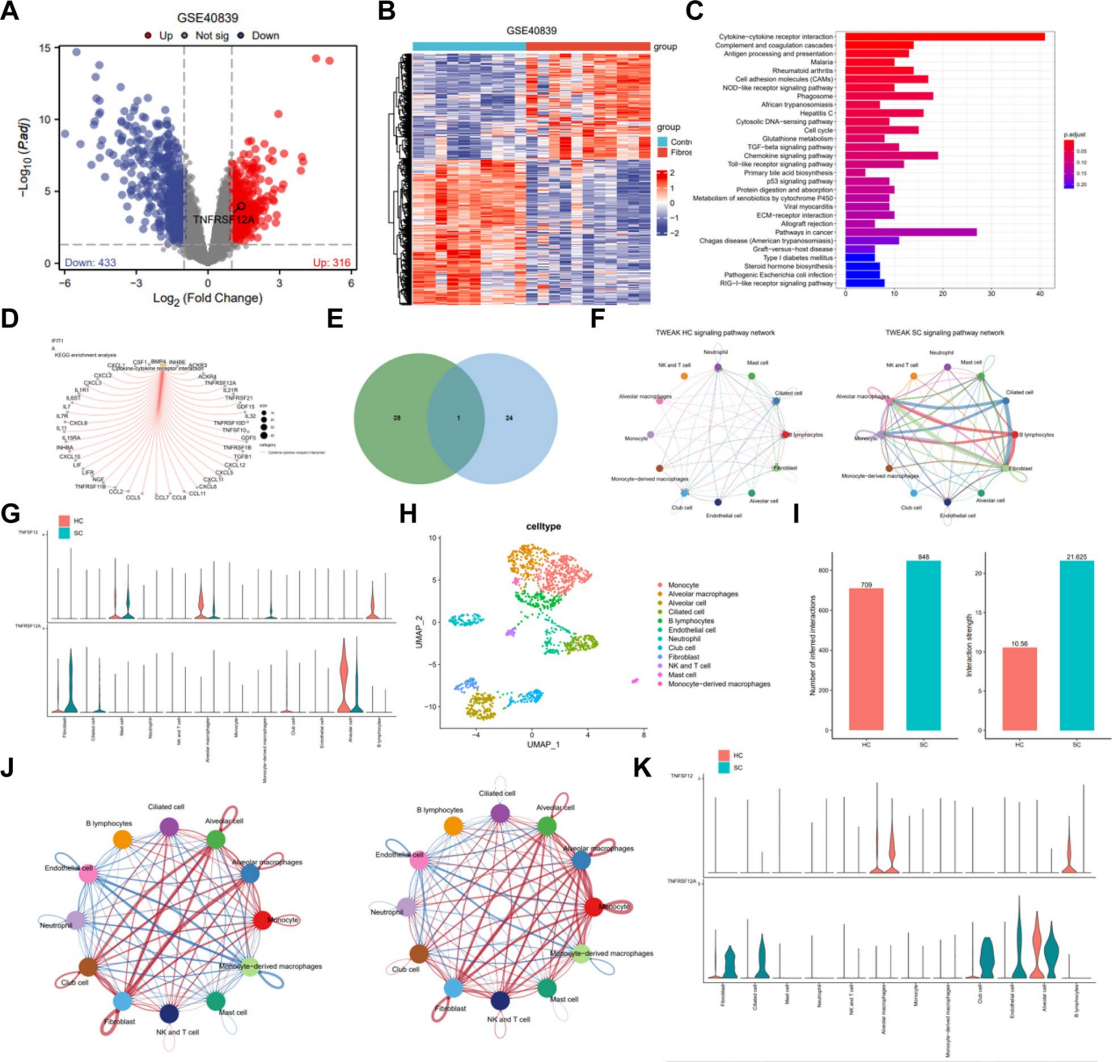
Differential expression analysis and functional enrichment of genes between normal and pulmonary fibrosis patient lung fibroblasts. **A**, **B** Volcano and heat maps depicting differentially expressed genes between normal control lung fibroblasts (Control group, n = 10) and fibroblast samples isolated from pulmonary fibrosis lung tissues (Fibrosis group, n = 11) in the GSE40839 dataset; **C** bar chart representing KEGG pathway enrichment analysis of differentially expressed genes; **D** network concept map of the cytokine-cytokine receptor interaction pathway (hsa04060) and its associated genes; **E** venn diagram showing the intersection of 40 pathway genes and 25 fibroblast receptor factors; **F** circle plot representing cell communication on the TWEAK pathway between healthy control lung tissue samples (HC group, n = 4) and severe COVID-19 patient lung tissue samples (SC group, n = 4), with thicker lines indicating stronger cell interactions; **G** violin plot presenting the expression of TNFRSF12A and TNFRSF12A in cells from healthy control lung tissue samples (HC group, n = 4) and severe COVID-19 patient lung tissue samples (SC group, n = 4), with red indicating HC group and blue indicating SC group; **H** cell annotation into 12 cell types based on the expression of known marker genes in a lung tissue sample from a severe COVID-19 patient after filtering; **I** bar chart comparing the number (left) and strength (right) of cell interactions on Secreted Signaling between healthy control lung tissue samples (HC group, n = 4) and severe COVID-19 patient lung tissue samples (SC group, n = 1), with red indicating a higher number and stronger interactions in the HC group, and blue indicating a higher number and stronger interactions in the SC group; **J** circle plot illustrating the comparison of the number (left) and strength (right) of cell interactions on Secreted Signaling between healthy control lung tissue samples (HC group, n = 4) and severe COVID-19 patient lung tissue samples (SC group, n = 1), with red lines indicating a higher number and stronger interactions in the SC group, and blue lines indicating a lower number and weaker interactions in the SC group, with thicker lines representing more significant cell interactions; **K** violin plot displaying the expression of TNFRSF12A and TNFRSF12A in cells from healthy control lung tissue samples (HC group, n = 4) and severe COVID-19 patient lung tissue samples (SC group, n = 1), with red indicating HC group and blue indicating SC group

Additionally, we obtained and downloaded the lung fibrosis-related microarray dataset GSE40839 from the GEO database. This dataset comprises 10 normal control lung fibroblast samples and 11 lung fibroblast samples isolated from patients with lung fibrosis. Differential analysis was conducted using a threshold of |logFC|> 1 and P < 0.05, identifying 316 upregulated genes and 433 downregulated genes (Fig. 5A, B; Table S4).

According to the KEGG pathway analysis results, the differentially expressed genes were primarily enriched in the Cytokine-cytokine receptor interaction (hsa04060) pathway (Fig. 5C). This finding aligns with our analysis of cellular communication. The cytokine-cytokine receptor interaction pathway comprises a total of 40 genes that are differentially expressed (Fig. 5D). By intersecting with 25 fibroblast receptor factors, we identified a single overlapping gene, specifically TNFRSF12A (Fig. 5E). Compared to lung fibroblasts from normal control samples, fibroblast samples obtained from pulmonary fibrosis showed an upregulation of TNFRSF12A (Fig. 5A).

The results above suggest that the fibroblast receptor factor TNFRSF12A might play a crucial role in the development of pulmonary fibrosis.

### Validation of alveolar macrophages and fibroblast interaction via TNFSF12-TNFRSF12A pathway in severe COVID-19 patients: implications for pulmonary fibrosis

The previous analysis results indicate that alveolar macrophages primarily influence fibroblasts through paracrine/autocrine signaling. The main signaling pathway involved in this process is TNFSF12 (TWEAK)-TNFRSF12A. Furthermore, we graphically represented the variations in cell communication between the HC group and the HC group TNFSF12-TNFRSF12A pathway using a circular plot.

Based on Fig. 5F, the SC group exhibited a greater intensity of cell interaction via the TWEAK pathway, including alveolar macrophages and fibroblasts, compared to the HC group. To further demonstrate the expression of genes in the TWEAK pathway in HC and SC cells, we observed TNFSF12 expression in alveolar macrophages in both the HC and SC groups. Additionally, TNFRSF12A showed higher expression in fibroblasts in the SC group (Fig. 5G), which aligns with the differential analysis findings from the GSE40839 dataset.

Finally, we obtained the scRNA-seq data (GSE148881) of a lung tissue sample from a severe COVID-19 patient and downloaded it from the CEO database as the testing dataset. It allowed us to validate the results of the cell communication analysis performed on the scRNA-seq above data. Similarly, we annotated a total of 1572 filtered cells from the GSE148881 dataset and categorized them into 12 distinct cell types: Neutrophil, NK and T cell, Alveolar macrophages, Alveolar cell, Monocyte, Club cell, Monocyte-derived macrophages, Endothelial cell, Mast cell, B lymphocytes, Ciliated cell, and Fibroblast (Fig. 5H).

The analysis of cell communication results reveals that, in terms of Secreted Signaling, the SC group displays a higher total number of cell interactions and greater interaction intensity than the HC group (Fig. 5I), consistent with the findings in Fig. 4A. Furthermore, the SC group demonstrates more frequent and tighter connections between Alveolar macrophages and Fibroblasts when compared to the HC group (Fig. 5J). TNFRSF12A is expressed in both the HC group and the SC group's Alveolar macrophages, with higher expression levels observed in the SC group. Notably, TNFRSF12A expression is also increased in the SC group's Fibroblasts (Fig. 5K).

Generally, the cell communication analysis results between the Testing and Training datasets are consistent. It further clarifies the communication between Alveolar Macrophages and Fibroblasts through the TNFSF12-TNFRSF12A pathway, ultimately promoting pulmonary fibrosis in severe COVID-19 patients.

### Alveolar macrophages promote fibroblast proliferation and pulmonary fibrosis via TNFSF12 signaling in SARS-CoV-2 exposure

**Fig. 6:**
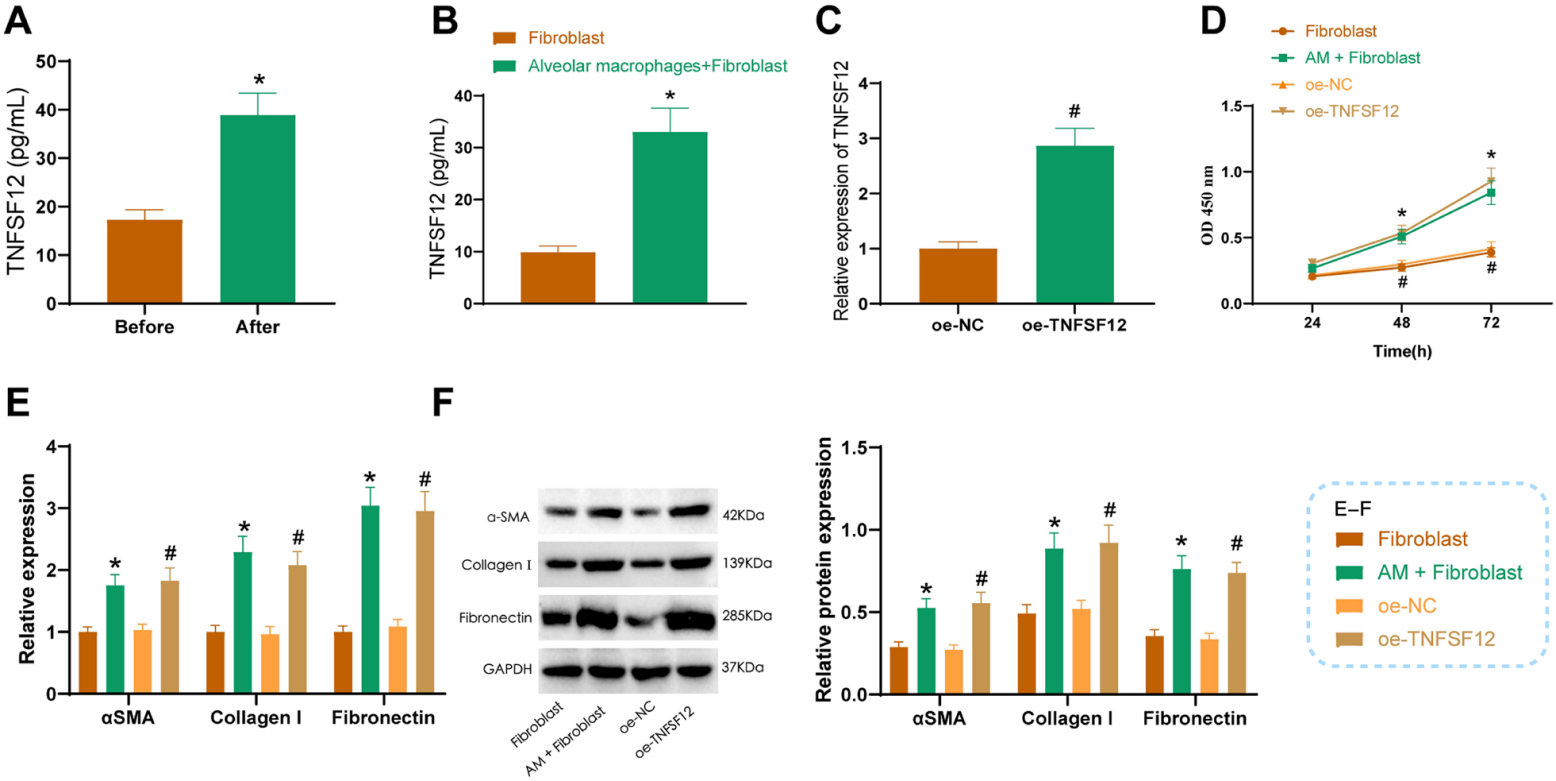
Alveolar macrophages promote fibroblast proliferation and fibrosis via secretion of TNFSF12. **A** ELISA measuring TNFSF12 levels in the cell culture supernatant of Alveolar macrophages before and after SARS-CoV-2-E treatment; **B** ELISA measuring TNFSF12 levels in the cell culture supernatant of Alveolar macrophages (AM) and Fibroblasts in co-culture; **C** qRT-PCR detecting TNFSF12 overexpression efficiency in Fibroblasts; **D** CCK-8 assay measuring Fibroblast cell proliferation; **E** qRT-PCR assessing mRNA expression of Extracellular Matrix (ECM) and fibrosis-related factors αSMA, Collagen I, and Fibronectin in Fibroblasts; **F** western blot examining protein expression of Extracellular Matrix (ECM) and fibrosis-related factors αSMA, Collagen I, and Fibronectin in Fibroblasts. *Indicates difference compared to the Fibroblast group, P < 0.05, #indicates difference compared to the oe-NC group, P < 0.05. Cell experiments were repeated three times

Based on the bioinformatics analysis presented above, we propose that alveolar macrophages communicate with fibroblasts via the TNFSF12-TNFRSF12A signaling pathway, thus promoting pulmonary fibrosis in severe COVID-19 patients. To investigate the communication mechanism between alveolar macrophages and fibroblasts, we co-cultured alveolar macrophages (AM) with SARS-CoV-2-E. After 24 h, we used Elisa to detect the TNFSF12 content in the supernatant of the AM cell culture. The results indicate an increase in the concentration of TNFSF12 in the culture supernatant of alveolar macrophages (AM) after 24 h of treatment with SARS-CoV-2-E compared to the pre-treatment levels (Fig. 6A).

In addition, we will co-culture alveolar macrophages (AM) and fibroblasts treated with SARS-CoV-2-E for 24 h. Then, we will measure the TNFSF12 level in the co-culture supernatant using ELISA. The results revealed an increase in the TNFSF12 content in the culture supernatant of the Alveolar macrophages + Fibroblast co-culture group compared to the fibroblast-alone culture group (Fig. 6B).

To examine the impact of TNFSF12 on fibroblast proliferation and fibrosis, we overexpressed TNFSF12 in fibroblasts and assessed its efficiency using qRT-PCR. The results indicate an increase in the expression level of TNFSF12 in fibroblasts transfected with TNFSF12 (Fig. 6C).

After inducing TNFSF12 overexpression in fibroblasts or co-culturing alveolar macrophages (AM) with fibroblasts, the proliferative capacity of fibroblast cells was assessed using CCK-8. The results demonstrated that the co-cultivation group of AM + Fibroblast exhibited higher proliferation ability in Fibroblast cells compared to the Fibroblast-alone cultivation group. Additionally, the oe-TNFSF12 group showed increased proliferation ability compared to the oe-NC group in Fibroblast cells (Fig. 6D).

The activation proliferation usually defines pulmonary fibrosis and extensive differentiation of fibroblasts, leading to an overproduction of extracellular matrix (ECM) proteins such as α-smooth muscle actin (αSMA), Collagen I, and Fibronectin [38, 39]. The expression of extracellular matrix (ECM) and fibrosis-related factors, namely αSMA, Collagen I, and Fibronectin was detected in fibroblasts using qRT-PCR and Western blot analysis.

The results demonstrated an increase in the expression of αSMA, Collagen I, and Fibronectin in Fibroblast cells in the AM + Fibroblast co-culture group compared to the Fibroblast monoculture group. Notably, the expression of αSMA, Collagen I, and Fibronectin in Fibroblast cells was also increased in the oe-TNFSF12 group compared to the oe-NC group (Fig. 6E, F).

In summary, co-culturing with alveolar macrophages or treating with TNFSF12 could enhance fibroblast proliferation and upregulate the expression of extracellular matrix (ECM) and fibrosis-related factors.

### TNFRSF12A modulation in fibroblasts: pivotal role in pulmonary fibrosis mediated by alveolar macrophage-derived TNFSF12

**Fig. 7:**
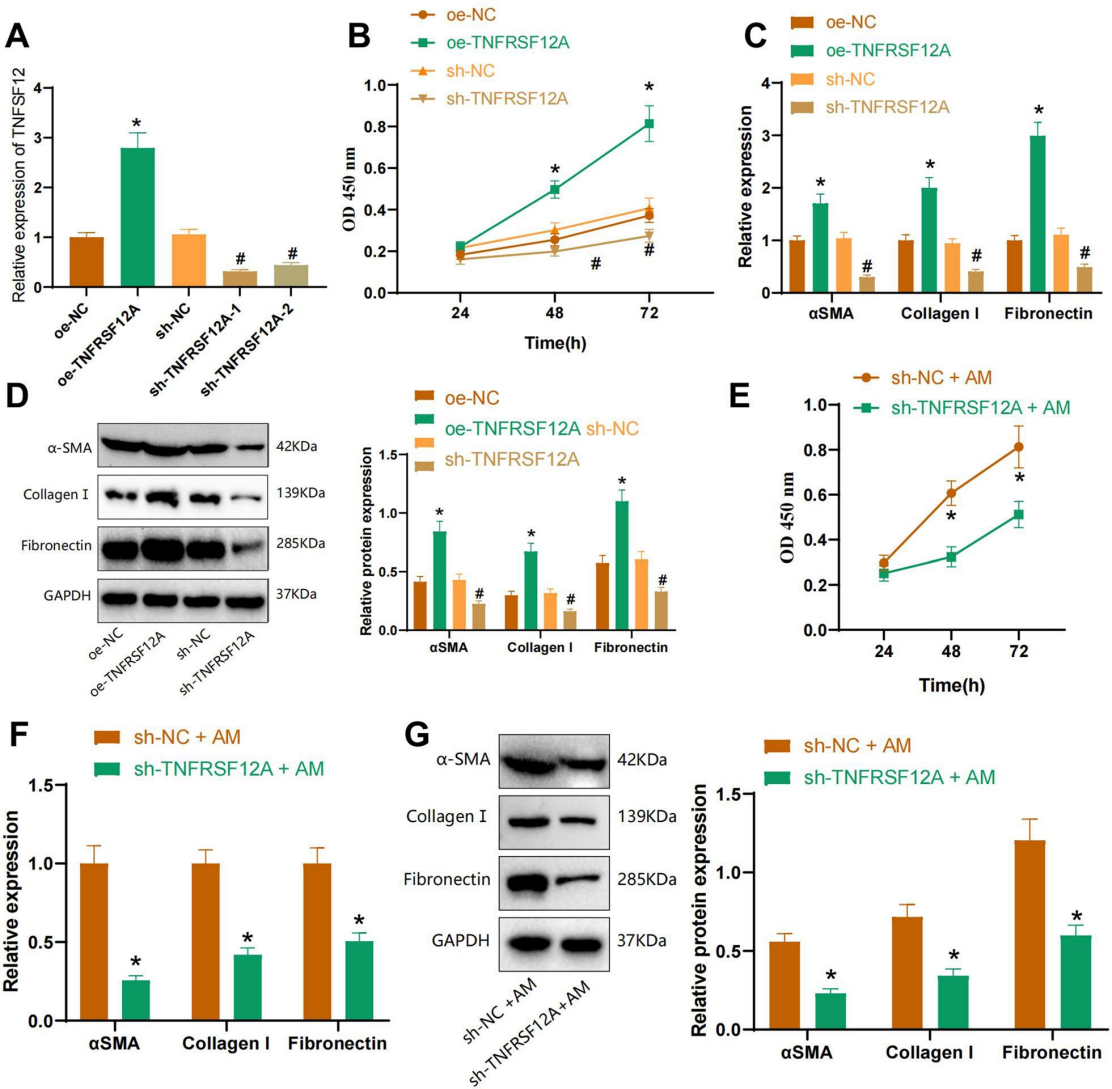
Alveolar macrophages enhance fibroblast proliferation and fibrosis by secreting TNFSF12 and binding to fibroblast receptor TNFRSF12A. **A** qRT-PCR assessing the silencing and overexpression efficiency of TNFRSF12A in Fibroblasts; **B** CCK-8 assay measuring Fibroblast cell proliferation; **C** qRT-PCR detecting mRNA expression of Extracellular Matrix (ECM) and fibrosis-related factors αSMA, Collagen I, and Fibronectin in Fibroblasts; **D** WB analysis of Extracellular Matrix (ECM) and fibrosis-related factors αSMA, Collagen I, and Fibronectin protein expression in Fibroblasts; **E** CCK-8 assay measuring Fibroblast cell proliferation; **F** qRT-PCR assessing mRNA expression of Extracellular Matrix (ECM) and fibrosis-related factors αSMA, Collagen I, and Fibronectin in Fibroblasts; **G** WB analysis of Extracellular Matrix (ECM) and fibrosis-related factors αSMA, Collagen I, and Fibronectin protein expression in Fibroblasts. *Indicates difference compared to the oe-NC group or sh-NC + AM group, P < 0.05, #indicates difference compared to the sh-NC group, P < 0.05. Cell experiments were repeated three times

The above results demonstrate that co-culturing with alveolar macrophages or treating with TNFSF12 could enhance fibroblast proliferation and fibrosis. To further confirm the influence of the fibroblast receptor TNFRSF12A on fibroblast proliferation and fibrosis, we manipulated TNFRSF12A expression in mouse lung fibroblasts through overexpression or knockdown. Initially, we assessed the efficiency of silencing and overexpression using qRT-PCR. We observed an increase in TNFRSF12A expression in fibroblasts from the oe-TNFRSF12A group compared to the oe-NC group. Moreover, sh-TNFRSF12A-1 and sh-TNFRSF12A-2 effectively silenced TNFRSF12A in fibroblasts compared to the sh-NC group, with sh-TNFRSF12A-1 exhibiting better results. Consequently, we selected it for subsequent experiments (sh-TNFRSF12A) (Fig. 7A).

The CCK-8 assay was performed to measure fibroblast cell proliferation. It was observed that the oe-TNFRSF12A group exhibited a higher ability of fibroblast cell proliferation compared to the oe-NC group. In contrast, the sh-TNFRSF12A group demonstrated a lower ability of fibroblast cell proliferation compared to the sh-NC group (Fig. 7B).

qRT-PCR and Western blot techniques were employed for evaluating the expression of extracellular matrix (ECM) and fibrosis-related factors in fibroblasts. The results demonstrate a substantial increase in the expression of αSMA, Collagen I, and Fibronectin in fibroblast cells in the oe-TNFRSF12A group compared to the oe-NC group. Furthermore, the sh-TNFRSF12A group reduced the expression of αSMA, Collagen I, and Fibronectin in fibroblast cells compared to the sh-NC group (Fig. 7C, D).

To investigate the impact of TNFSF12 secreted by alveolar macrophages on fibroblast fibrosis through the fibroblast receptor TNFRSF12A, we co-cultured fibroblast cells with alveolar macrophages after knocking down TNFRSF12A. Given that pulmonary fibrosis typically manifests as activated proliferation of fibroblast cells, we initially assessed fibroblast cell proliferation using the CCK8 assay. Our findings revealed a significant decrease in the proliferation capacity of fibroblast cells in the sh-TNFRSF12A + AM group compared to the sh-NC + AM group (Fig. 7E).

Expression of extracellular matrix (ECM) and fibrosis-related factors in fibroblasts was examined using qRT-PCR and Western blot. The results demonstrated a reduction in the levels of αSMA, Collagen I, and Fibronectin in fibroblasts from the sh-TNFRSF12A + AM group compared to the sh-NC + AM group (Fig. 7F, G).

The results indicate that the overexpression or silencing of TNFRSF12A could promote or inhibit fibroblast cell proliferation and fibrosis. Additionally, silencing TNFRSF12A could reverse the pro-fibrotic effect of alveolar macrophages. This finding suggests that alveolar macrophages initiate pulmonary fibrosis by releasing TNFSF12 and interacting with the fibroblast receptor TNFRSF12A.

## Discussion

The incidence of pulmonary fibrosis is a significant concern among the complications associated with severe COVID-19, as it has a direct impact on patients' prognosis and long-term quality of life [40]. Pulmonary fibrosis occurs due to the excessive scarring of lung tissue, which results in impaired respiratory function [41]. The existing research has revealed the central role that alveolar macrophages play in this process, particularly in their interactions with fibroblasts [42]. Recent studies have indicated that alveolar macrophages establish communication with fibroblasts through the TNFSF12-TNFRSF12A pathway, which may potentially expedite the progression of pulmonary fibrosis [43]. The close interaction among these cells generates novel ideas and identifies potential targets for future targeted therapy [44].

This study examines single-cell RNA sequencing data from lung tissues of critically ill COVID-19 patients and reveals that, in comparison to the healthy control group, the patient group may exhibit lung alveolar macrophages originating primarily from monocytes. Cell transformation is not only reflected in the increase in quantity, but also in significant changes in antigen presentation function [45]. Importantly, our analysis of cellular communication has revealed that cells in the lung tissue of severe COVID-19 patients primarily communicate with each other through Secreted Signaling and Cell–Cell Contact mechanisms. The interaction between alveolar macrophages and fibroblasts is especially notable in these cases [46]. This specific interaction is mediated via the TNFSF12-TNFRSF12A pathway and is regarded as a crucial mechanism in promoting pulmonary fibrosis. In order to validate this finding, we performed a series of in vitro cell experiments. The experimental results demonstrated that co-culturing with alveolar macrophages or treating with TNFSF12 substantially enhanced fibroblast cell proliferation and resulted in increased expression of extracellular matrix (ECM) and fibrosis-related factors. Furthermore, by manipulating the expression or silence of TNFRSF12A, we have unequivocally established the pivotal role of this pathway in regulating both fibroblast cell proliferation and fibrosis. It is important to note that silencing TNFRSF12A not only inhibits the proliferation and fibrosis of fibroblasts, but also reverses the promoting effect of alveolar macrophages [47]. In summary, our research elucidates for the first time the crucial mechanism of 'communication' between alveolar macrophages and fibroblasts via the TNFSF12-TNFRSF12A pathway, emphasizing its central role in pulmonary fibrosis in severe COVID-19 patients.

Notably, this research offers a novel perspective for examining the mechanism of pulmonary fibrosis in critically ill COVID-19 patients. Although the crucial role of alveolar macrophages in disease progression is widely recognized, this study provides additional insight into their distinct ‘communication’ with fibroblasts via the TNFSF12-TNFRSF12A pathway, which challenges previous understanding [48]. It is worth noting that the utilization of single-cell RNA sequencing technology in this study enables us to investigate the communication mechanisms between cells at the individual cell level [49]. The utilization of this technology not only offers us comprehensive data support, but also guarantees the reliability and accuracy of research findings [50]. Overall, comparing our research findings with those of other studies strengthens our confidence in the new insights we have generated, highlighting the distinctiveness of single-cell RNA sequencing in uncovering intercellular interactions.

## Conclusion

**Fig. 8:**
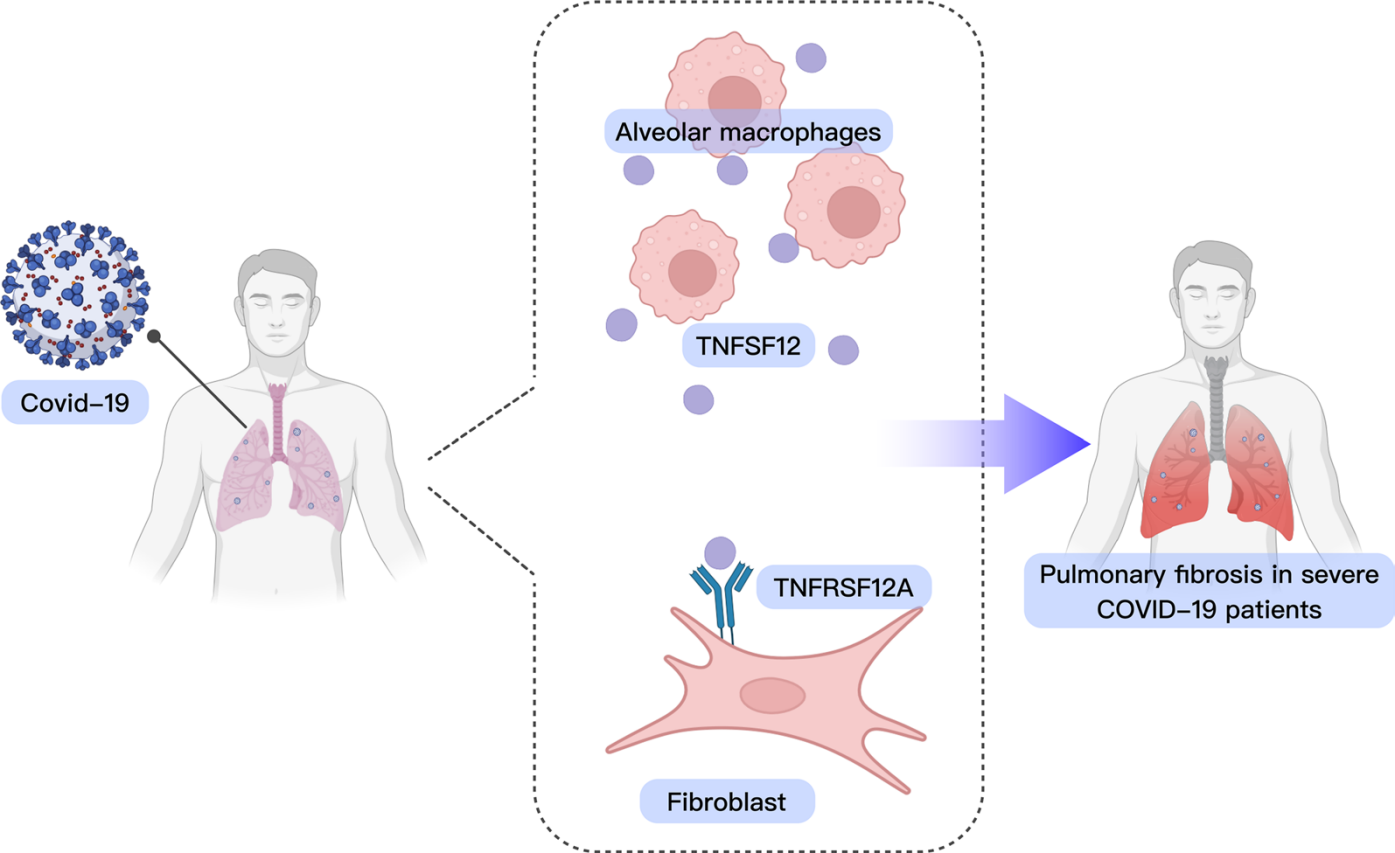
Mechanism illustration of alveolar macrophages promoting severe COVID-19 patient lung fibrosis via the TNFSF12-TNFRSF12A pathway

This study highlights the crucial role of communication between alveolar macrophages and fibroblasts via the TNFSF12-TNFRSF12A pathway in the development of pulmonary fibrosis (Fig. 8). This significant discovery establishes a robust scientific foundation for the future development and optimization of treatment strategies for severe COVID-19 patients. The significance of this lies in the understanding that this pathway has the potential to assist the medical community in offering more precise and targeted treatment options for critically ill COVID-19 patients. However, it is important to acknowledge certain limitations of this study, including the small sample size and potential variations in experimental conditions when compared to the clinical setting. In the future, additional extensive and in-depth research will further validate these findings and investigate the potential of the TNFSF12-TNFRSF12A pathway in clinical applications.

### Supplementary Information

Supplementary Material 1. Figure S1. Quality Control and Variance Analysis of scRNA-seq Data. (A) Quality control of cell sources from lung tissue samples of 4 severe COVID-19 patients; (B) Correlation between nCount and percent.mt (top) and between nCount and nFeature (bottom) in cells from lung tissue samples of 4 severe COVID-19 patients; (C) Variance analysis selecting highly variable genes in cells from lung tissue samples of 4 severe COVID-19 patients; (D) Quality control of cell sources from lung tissue samples of 4 healthy controls; (E) Correlation between nCount and percent.mt (top) and between nCount and nFeature (bottom) in cells from lung tissue samples of 4 healthy controls; (F) Variance analysis selecting highly variable genes in cells from lung tissue samples of 4 healthy controls. In panels A and D, the three scatter plots represent the number of genes per cell (nFeature_RNA), the number of RNA molecules per cell (nCount_RNA), and the percentage of mitochondrial genes (percent.mt); in panels C and F, red dots represent highly variable genes, and black dots represent non-variable genes.

Supplementary Material 2. Figure S2. Principal Component Analysis of scRNA-seq Data. (A) Cellular cycle status of cells sourced from lung tissue samples of 4 severe COVID-19 patients and 4 healthy controls, with S.Score representing the S phase and G2M.Score representing the G2M phase; (B) Cell clustering based on principal component analysis; (C) Heatmap of gene expression of the top genes contributing to the first two principal components; (D) Point plot illustrating the gene composition of the first two principal components; (E) Comparison of p-values for each principal component using the JackStrawPlot function; (F) Determination of the principal components to be used for subsequent analysis by examining the change in variance and identifying inflection points, where important PCs have higher standard deviation; (G) UMAP analysis clustering all cells into 20 cell clusters, with each color representing a cluster; (H) Further annotation of the 5 major cell classes into 12 cell types based on the expression of known marker genes.

Supplementary Material 3. Figure S3. Cellular Communication Analysis in Lung Tissue Samples of Severe COVID-19 Patients and Healthy Controls. (A) Composition of CellChatDB in humans; (B-D) Heatmaps comparing the number of cell interactions (left) and interaction strength (right) between lung tissue samples from healthy controls (HC group, n=4) and severe COVID-19 patients (SC group, n=4), with red squares indicating more interactions and stronger interactions in the SC group, while blue squares indicate fewer interactions and weaker interactions in the SC group. (B) Secreted Signaling; (C) ECM-Receptor; (D) Cell-Cell Contact.

Supplementary Material 4.

Supplementary Material 5.

Supplementary Material 6.

Supplementary Material 7.

## Data Availability

The datasets used and/or analysed during the current study are available from the corresponding author on reasonable request.

## Abbreviations

COVID-19: Coronavirus disease 2019
PM: PubMed ID (used to reference specific articles)
scRNA-seq: Single-cell RNA sequencing
GEO: Gene expression omnibus
UMI: Unique molecular identifier
PCA: Principal component analysis
UMAP: Uniform manifold approximation and projection
GO: Gene ontology
KEGG: Kyoto Encyclopedia of Genes and Genomes
CCK-8: Cell counting kit-8
ELISA: Enzyme-linked immunosorbent assay
RT-qPCR: Real-time quantitative polymerase chain reaction
SDS-PAGE: Sodium dodecyl sulfate–polyacrylamide gel electrophoresis
PVDF: Polyvinylidene difluoride
HRP: Horseradish peroxidase
α-SMA: Alpha-smooth muscle actin
GAPDH: Glyceraldehyde-3-phosphate dehydrogenase
