# Supplementary figures and images for: Communication between alveolar macrophages and fibroblasts via the TNFSF12-TNFRSF12A pathway promotes pulmonary fibrosis in severe COVID-19 patients
Source: J Transl Med. 2024 Jul 29;22:698. doi: 10.1186/s12967-024-05381-7 (PMC11287943; doi:10.1186/s12967-024-05381-7)

A

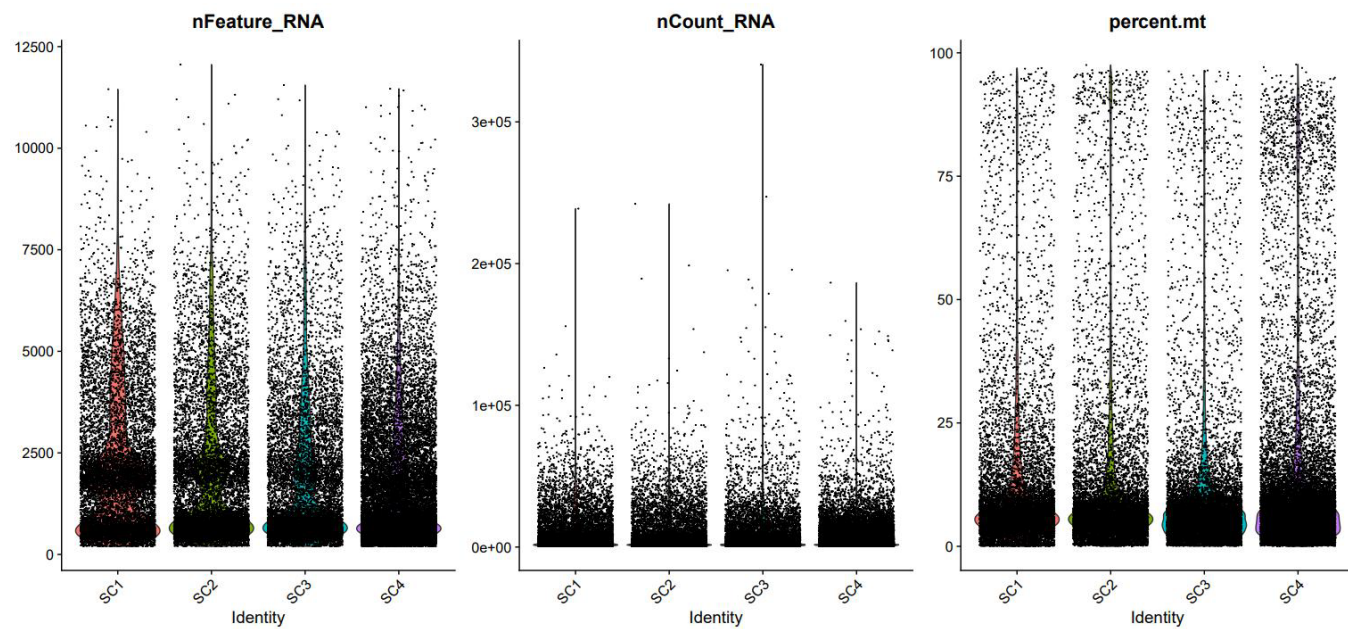

B

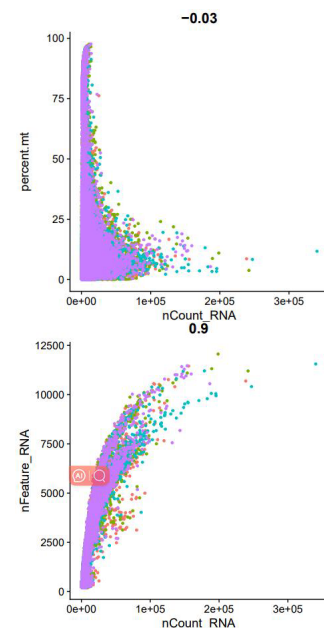

C

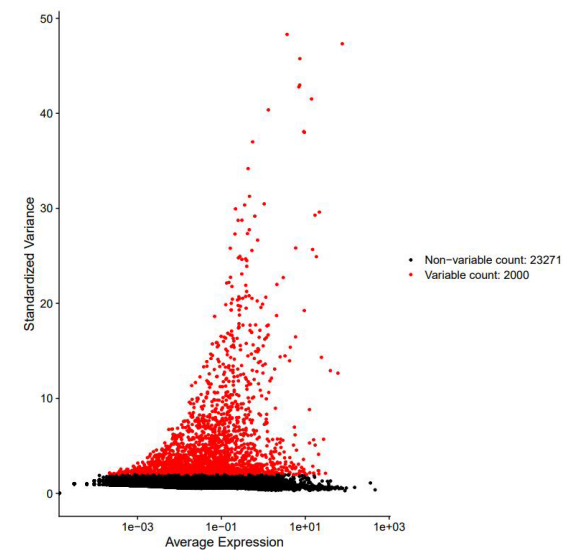

D

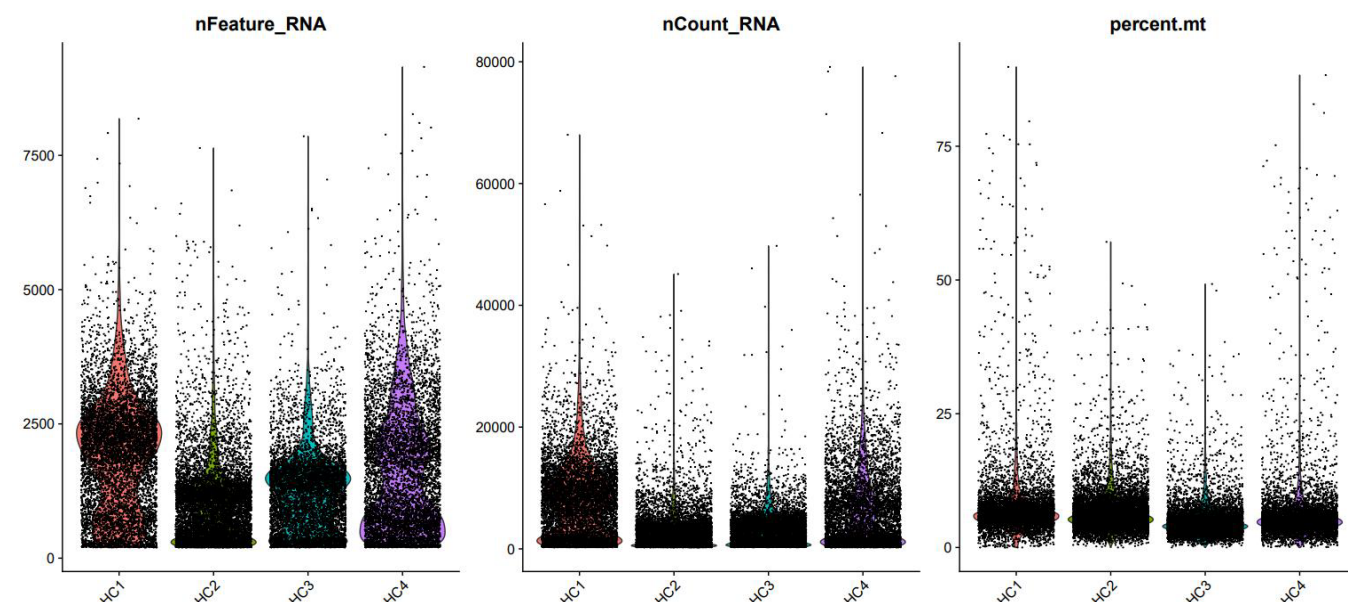

E

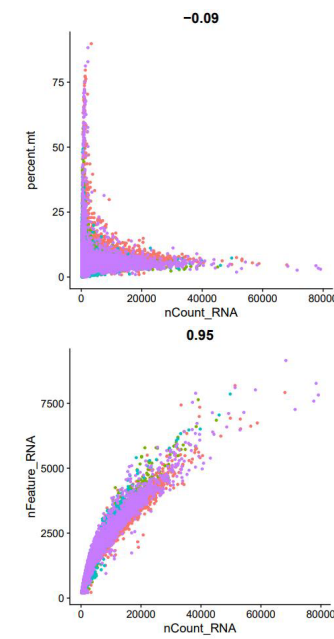

F

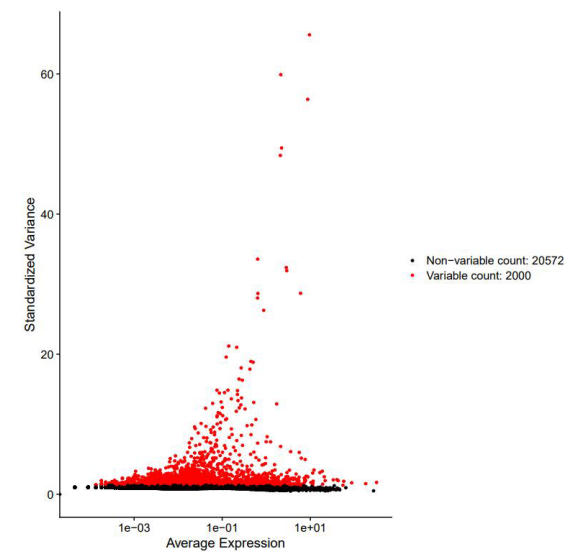

Supplement: Supplementary file 1 — Supplementary Material 1. Figure S1. Quality Control and Variance Analysis of scRNA-seq Data. (A) Quality control of cell sources from lung tissue samples of 4 severe COVID-19 patients; (B) Correlation between nCount and percent.mt (top) and between nCount and nFeature (bottom) in cells from lung tissue samples of 4 severe COVID-19 patients; (C) Variance analysis selecting highly variable genes in cells from lung tissue samples of 4 severe COVID-19 patients; (D) Quality control of cell sources from lung tissue samples of 4 healthy controls; (E) Correlation between nCount and percent.mt (top) and between nCount and nFeature (bottom) in cells from lung tissue samples of 4 healthy controls; (F) Variance analysis selecting highly variable genes in cells from lung tissue samples of 4 healthy controls. In panels A and D, the three scatter plots represent the number of genes per cell (nFeature_RNA), the number of RNA molecules per cell (nCount_RNA), and the percentage of mitochondrial genes (percent.mt); in panels C and F, red dots represent highly variable genes, and black dots represent non-variable genes. [file 12967_2024_5381_MOESM1_ESM.pdf]

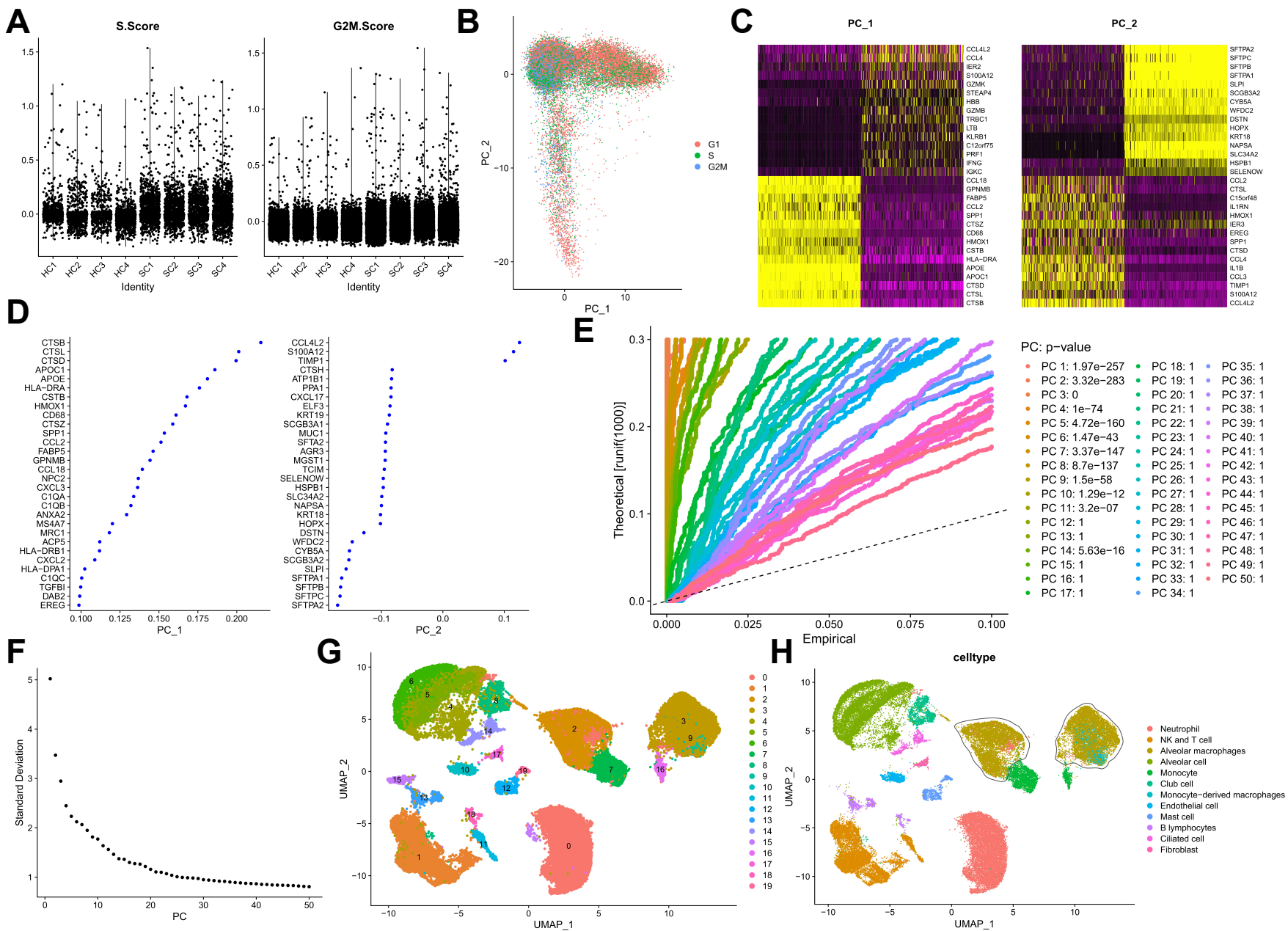

Supplement: Supplementary file 2 — Supplementary Material 2. Figure S2. Principal Component Analysis of scRNA-seq Data. (A) Cellular cycle status of cells sourced from lung tissue samples of 4 severe COVID-19 patients and 4 healthy controls, with S.Score representing the S phase and G2M.Score representing the G2M phase; (B) Cell clustering based on principal component analysis; (C) Heatmap of gene expression of the top genes contributing to the first two principal components; (D) Point plot illustrating the gene composition of the first two principal components; (E) Comparison of p-values for each principal component using the JackStrawPlot function; (F) Determination of the principal components to be used for subsequent analysis by examining the change in variance and identifying inflection points, where important PCs have higher standard deviation; (G) UMAP analysis clustering all cells into 20 cell clusters, with each color representing a cluster; (H) Further annotation of the 5 major cell classes into 12 cell types based on the expression of known marker genes. [file 12967_2024_5381_MOESM2_ESM.pdf]
