# Supplementary material for: Communication between alveolar macrophages and fibroblasts via the TNFSF12-TNFRSF12A pathway promotes pulmonary fibrosis in severe COVID-19 patients
Source: J Transl Med. 2024 Jul 29;22:698. doi: 10.1186/s12967-024-05381-7 (PMC11287943; doi:10.1186/s12967-024-05381-7)
Supplement: Supplementary file 3 — Supplementary Material 3. Figure S3. Cellular Communication Analysis in Lung Tissue Samples of Severe COVID-19 Patients and Healthy Controls. (A) Composition of CellChatDB in humans; (B-D) Heatmaps comparing the number of cell interactions (left) and interaction strength (right) between lung tissue samples from healthy controls (HC group, n=4) and severe COVID-19 patients (SC group, n=4), with red squares indicating more interactions and stronger interactions in the SC group, while blue squares indicate fewer interactions and weaker interactions in the SC group. (B) Secreted Signaling; (C) ECM-Receptor; (D) Cell-Cell Contact. [file 12967_2024_5381_MOESM3_ESM.pdf]

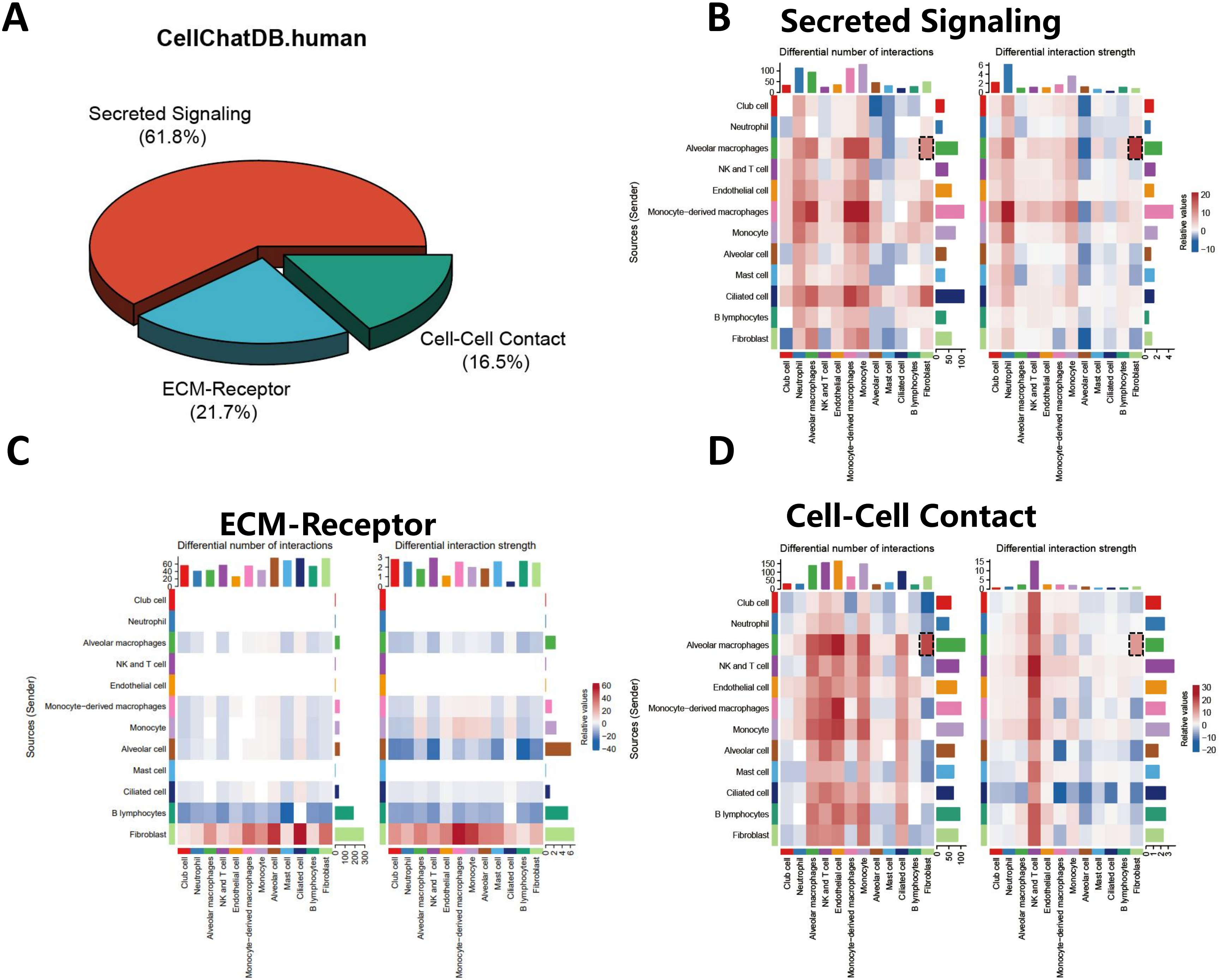

A
CellChatDB.human
Secreted Signaling (61.8%)
ECM-Receptor (21.7%)
Cell-Cell Contact (16.5%)
B
Secreted Signaling
Differential number of interactions
Differential interaction strength
Sources (Sender)
Relative values
Club cell
Neutrophil
Alveolar macrophages
NK and T cell
Endothelial cell
Monocyte-derived macrophages
Monocyte
Alveolar cell
Mast cell
Ciliated cell
B lymphocytes
Fibroblast
C
ECM-Receptor
Differential number of interactions
Differential interaction strength
Sources (Sender)
Relative values
D
Cell-Cell Contact
Differential number of interactions
Differential interaction strength
Sources (Sender)
Relative values
