# Supplementary material for: Communication between alveolar macrophages and fibroblasts via the TNFSF12-TNFRSF12A pathway promotes pulmonary fibrosis in severe COVID-19 patients
Source: J Transl Med. 2024 Jul 29;22:698. doi: 10.1186/s12967-024-05381-7 (PMC11287943; doi:10.1186/s12967-024-05381-7)
Supplement: Supplementary file 4 — Supplementary Material 4. [file 12967_2024_5381_MOESM4_ESM.docx]

**Supplemental table 1. GEO Sample information of the dataset**

| Dataset | Sample | Type |
| --- | --- | --- |
| GSE122960 | Healthy control lung tissue samples (HC group, n=4) | scRNA-seq |
| GSE149878 | Lung tissue samples from severe COVID-19 patients (SC group, n=4) | scRNA-seq |
| GSE148881 | Lung tissue samples from severe COVID-19 patients (SC group, n=1) | scRNA-seq |
| GSE40839 | Fibroblast samples isolated from normal control lung fibroblasts (Control group, n=10) and pulmonary fibrosis lung tissue (Fibrosis group, n=11) | Array |

Note: HC represents health control, SC represents severe COVID-19, and scRNA seq represents single cell RNA sequencing.
